# Functional and Regulatory Characterization of Three AMTs in Maize Roots

**DOI:** 10.3389/fpls.2020.00884

**Published:** 2020-06-26

**Authors:** Dong-Li Hao, Jin-Yan Zhou, Shun-Ying Yang, Ya-Nan Huang, Yan-Hua Su

**Affiliations:** State Key Laboratory of Soil and Sustainable Agriculture, Institute of Soil Science, Chinese Academy of Sciences, Nanjing, China

**Keywords:** maize, ammonium transporter, electrophysiology, pH regulation, phosphorylation regulation, physiological significance

## Abstract

Maize grows in nitrate-dominated dryland soils, but shortly upon localized dressing of nitrogen fertilizers, ammonium is retained as a noticeable form of nitrogen source available to roots. Thus in addition to nitrate, the absorption of ammonium can be an important strategy that promotes rapid plant growth at strong nitrogen demanding stages. The present study reports the functional characterization of three root-expressed ammonium transporters (AMTs), aiming at finding out functional and regulatory properties that correlate with efficient nitrogen acquisition of maize. Using a stable electrophysiological recording method we previously established in *Xenopus laevis* oocytes that integrates the capture of currents in response to voltage ramps with onsite stability controls, we demonstrate that all three ZmAMT1s engage NH_4_^+^ uniporting as ammonium uptake mechanisms. The *K*_*m*_ value for ZmAMT1.1a, 1.1b, or ZmAMT1.3 is, respectively, 9.9, 15.6, or 18.6 μM, indicating a typical high-affinity transport of NH_4_^+^ ions. Importantly, the uptake currents of these ZmAMT1s are markedly amplified upon extracellular acidification. A pH drop from 7.4 to 5.4 results in a 140.8%, 64.1% or a 120.7% increase of ammonium uptake activity through ZmAMT1.1a, 1.1b, or ZmAMT1.3. Since ammonium uptake by plant roots accompanies a spontaneous acidification to the root medium, the functional promotion of ZmAMT1.1a, 1.1b, and ZmAMT1.3 by low pH is in line with the facilitated ammonium uptake activity in maize roots. Furthermore, the expression of the three *ZmAMT1* genes is induced under ammonium-dominated conditions. Thus we describe a facilitated ammonium uptake strategy in maize roots by functional and expression regulations of ZmAMT1 transporters that may coordinate with efficient acquisition of this form of nitrogen source when available.

## Introduction

Maize grows in nitrate-dominated dryland soils. Throughout the lifespan of maize, the largest nutrition/fertilizer demand occurs at the large flare stage (with 11−12 leaves) (Cui et al., 2009). To gain high yield, nitrogen topdressing is usually conducted during this stage in agricultural production. Such practice can quickly lead to ammonium dominating the maize-growing soils. It is thus tempting to speculate that, to meet the strongest nitrogen demand, maize has to efficiently uptake and utilize the dominant inorganic nitrogen source, ammonium, in soils, at least during the large flare stage post-fertilization. When supplied at equivalent concentrations, ammonium can support a similar or greater maize growth compared with nitrate (Engels and Marschner, 1993; Wang et al., 2019; Zhang et al., 2019), indicating that maize has a strong ammonium absorption system. Ammonium uptake in plants is undertaken by ammonium transporters (AMTs) (Von Wirén and Merrick, 2004; Ludewig et al., 2007; Loqué et al., 2009).

The ammonium transport activity of plant AMTs is regulated by many external environmental factors. It has been reported that the activities of the wheat AMT TaAMT1;1 (Søgaard et al., 2009) and the common bean AMT PvAMT1;1 (Ortiz-Ramirez et al., 2011) are facilitated by low pH, whereas that of the tomato AMTs LeAMT1;1 (Ludewig et al., 2002) and LeAMT1;2 (Ludewig et al., 2003), the Arabidopsis AMT AtAMT1;1 (Loqué et al., 2009), and the rice AMT OsAMT1;1 (Yang et al., 2015) are insensitive to pH changes. Additionally, the functional switch of plant AMTs is dominated by the phosphorylation status of a threonine (T460) at its carboxyl-terminus (Straub et al., 2017; Beier et al., 2018). Substitution of this T460 site from AtAMT1;1 or its counterparts from other plant AMTs, with an amino acid alanine, to mimic the de-phosphorylated status of this position, leads to a functional AMT. However, substitution of this site to the amino acid glutamate, to mimic the phosphorylated status of this position, results in a loss-of-function in the AMT (Loqué et al., 2007; Neuhäuser et al., 2007; Yuan et al., 2013; Guo et al., 2018; Wu et al., 2019). This threonine site is rapidly phosphorylated *in planta* upon high ammonium exposure, resulting in a rapid shut-off of the ammonium absorption capacity mediated by AMTs, preventing ammonium toxicity (Lanquar et al., 2009). In contrast, another member of the AMT/MEP/Rh family, Mep2 from *Candida albicans*, is activated when the carboxyl-terminal serine is in its phosphorylated status, and is inactivated when this serine is in its de-phosphorylated status (Van Den Berg et al., 2016). The different functional regulation strategies among AMTs are most likely associated with the nitrogen requirements of plants.

Nitrogen topdressing at the jointing stage or large flare stage is an important guarantee for high yield of maize, resulting in ammonium nitrogen dominance in soils for a short time. At least in this case, efficient ammonium uptake carried by AMTs is seemingly necessary for the nitrogen demand of maize. Whether the AMTs engage functional or regulatory properties that are adapted to the efficient absorption of ammonium nitrogen, under the ammonium-dominated conditions, remains largely unclear. Therefore, in this study, all three functional AMTs from maize roots (Gu et al., 2013) were firstly investigated using the two-electrode voltage clamp technique. Through analysis of their response to varied ammonium concentration, nitrate co-existence, glutamine, phosphorylation as well as response to pH changes, the functional and regulatory properties of ZmAMTs were determined. Then, through comparison of the biomass and nitrogen contents upon the different forms of nitrogen treatments under hydroponic conditions, the growth-supporting effect of ammonium that compared with nitrate was identified. Thereafter, through comparison of the ammonium uptake rate upon changed Ca^2+^, nitrate, pH in solutions, the characterization of ammonium uptake regulation in maize roots was investigated. From the combination of the findings in the heterologous oocytes expression system and *in planta* maize roots, it is speculated that maize could achieve efficient absorption of ammonium nitrogen by virtue of acidification caused by an ammonium-dominated soil environment (such as after nitrogen fertilizer application).

## Materials and Methods

### Maize Growth Experiment

Maize seeds (variety: Zhengdan 958) were soaked in water for 5 days and then the germinated seedlings were transferred to a nutrient solution and pre-cultured for 14 days. The nutrient solution was composed of 0.5 mM NH_4_NO_3_, 0.3 mM KH_2_PO_4_, 0.35 mM K_2_SO_4_, 1 mM CaCl_2_, 1 mM MgSO_4_.7H_2_O, 20 μM EDTA-Fe, 20 μM H_3_BO_3_, 9 μM MnCl_2_.4H_2_0, 0.77 μM ZnSO_4_.7H_2_O, 0.32 μM CuSO_4_.5H_2_O, and 0.39 μM Na_2_MoO_4_.2H_2_O. Uniform seedlings were thereafter selected to receive treatments with changed nitrogen forms: nitrate only (1 mM NaNO_3_), ammonium only (1 mM NH_4_Cl), or a mixture of ammonium and nitrate (0.5 mM NH_4_NO_3_) in nutrient solution. The maize seedlings were cultivated in a growth room with a 14-h-light (28°C)/10-h-dark (25°C) photoperiod, and the relative humidity was adjusted to approximately 70%. The light intensity was 400 μmol m^–2^ s^–1^. The pH of the nutrient solution was adjusted to 5.8, and the nutrient solution was refreshed every 3 days. After 10 days of treatment, the plants were harvested and separated into roots and shoots. The dry weight was measured, and the total nitrogen content was determined by the Kjeldahl method (Sparks et al., 1996) after digestion with H_2_SO_4_-H_2_O_2_.

### Ammonium Uptake by Seedling Roots

Maize seeds (variety: Zhengdan 958) were firstly soaked for 5 days in water. The germinated maize seedling was then pre-cultured for 14 days in a nutrient solution containing 0.5 mM NH_4_NO_3_, followed by a nitrogen-free nutrient solution treatment for 3 days. The composition of the nutrient solution used was described in section “Maize Growth Experiment,” and the composition of the nitrogen-free nutrient solution was similar to it, only without 0.5 mM NH_4_NO_3_. After 3 days of nitrogen starvation, the roots were soaked in 0.1 mM CaSO_4_ for 5 min and then rinsed with distilled water. Finally, the roots were immersed in various ammonium uptake solutions containing 0.1 mM CaSO_4_ (pH 5.5), but with changes in ammonium concentration (added as NH_4_Cl), or introduction of Ca^2+^ (added as CaCl_2_) or nitrate (added as NaNO_3_) as desired. For the determination of ammonium uptake rate at different pH values, the pH of the ammonium uptake solutions was adjusted to 4.5 or 6.5. Each maize seedling was carefully placed in a 250 mL beaker, and its roots were immersed in 200 mL of ammonium uptake solution. Due to the scattered distribution of maize roots, such practice is absolutely necessary to avoid damage to maize roots. The outside of the beaker was covered with tin foil to prevent the influence of light on the roots. The ammonium uptake test started in the light cycle (9:30 am) and was sustained for 1 h. The residual fluid was then collected, and the fresh weight of the corresponding roots was simultaneously measured. The concentration of ammonium nitrogen retained in the ammonium uptake solution was determined by Nessler’s reagent method according to the manufacturer’s instructions (Shanghai Yuanye Biological Technology Company, China). The ammonium absorption rate was determined with the following equation:

Ammonium absorption rate = (initial concentration - sample concentration) × volume/(absorption time × root weight)

For ammonium uptake kinetics studies, the applied initial concentration of ammonium was 20, 35, 50, 100, or 250 μM. For other uptake assays, the initial concentration of ammonium was 100 μM. The ammonium retained after ammonium uptake test was referred to as sample concentration. The volume of ammonium uptake solution used was 200 mL.

### Real-Time Quantitative PCR

After 5 days of soak in water, the geminated maize seedlings were pre-cultured for 14 days in the nutrient solution containing 0.5 mM NH_4_NO_3_ (the composition of which has been described in section “Maize Growth Experiment”). Uniform seedlings were then subjected to treatments with changing nitrogen forms: nitrate only (1 mM NaNO_3_), ammonium only (1 mM NH_4_Cl) or ammonium nitrate (0.5 mM NH_4_NO_3_). Root samples were harvested at 0, 3, 6, 12, 24, and 72 h after the nitrogen treatments began and stored at −80°C. RNA was extracted from maize root samples according to the manufacturer’s instructions using TRIzol reagent (Invitrogen). One microgram of RNA was reverse-transcribed into cDNA using a kit (TaKaRa, Cat # RR047A). The quantitative PCR was performed with a C1000 Thermal Cycler CFX96 Real-Time System (Bio-Rad) instrument using SYBR Green Perfect mix (TaKaRa, Cat # RR420A). The quantitative PCR program started with initial denaturation (95°C for 30 s), followed by 40 cycles of 95°C for 5 s, 60°C for 10 s, 72°C for 15 s. The gene expression abundances of *ZmAMT1s* were calculated using the 2^–Δ^ ^*CT*^ method with *ZmACT1* (J01238) as an endogenous control. The quantitative PCR primers used are listed in Supplementary Table 1.

### Vector Constructions

RNA was extracted according to the manufacturer’s instructions using TRIzol reagent (Invitrogen) from maize roots being pre-cultivated for 14 days in the nutrient solution containing 0.5 mM NH_4_NO_3_ (the composition of which has been described in section “Maize Growth Experiment”), and then was reverse-transcribed into cDNA using a kit (TaKaRa, Cat # 6110A). Three *ZmAMT1* genes were amplified by PCR using cDNA as a template and then were cloned into the oocyte expression vector pCI by homologous recombination using a kit (Vazyme, Cat # C112). The PCR program started with initial denaturation (95°C for 5 min) followed by 35 cycles of 95°C for 30 s, 60°C for 30 s, 72°C for 100 s, finally with one time 72°C for 10 min. Phosphorylation site mutants were obtained using overlapping PCR and were subsequently cloned into the pCI vector. The sequences were verified by sequencing through the Beijing Genomics Institution (BGI, China). AtAMT1;3 and its T464D mutant vector constructs were cloned using a similar process to those of maize ZmAMT1s, except RNA was extracted from the roots of 6-week-old Arabidopsis thaliana. The primers used for vector constructions are listed in Supplementary Table 2.

### Electrophysiology

The preparation and injection of the *Xenopus lavies* oocytes were performed as previously described (Hao et al., 2016). Each oocyte received 60 ng (1 μg/1 μL, 60 nL) of plasmid DNA containing *ZmAMT1*-pCI, *AtAMT1;3*-pCI or their mutants, and was cultured at 19°C in a constant-temperature incubator for 3−4 days. Oocytes injected with an equal volume (60 nL) of H_2_O were used as controls. The response of these AMTs-expressing oocytes or the control ones, to ammonium concentration serials or various imposed conditions was then detected by the two-electrode voltage clamp technique. The basal bath solution contained 100 mM NaCl, 2 mM CaCl_2_, 2 mM MgCl_2_, and 4 mM Tris (the pH was adjusted to 7.4 with MES). For the pH 5.4 solution, 4 mM Tris was replaced with 4 mM MES, and the pH was adjusted using Tris. Ammonium and other components were added or changed as required. Ammonium and calcium were added as chloride salts. Nitrate was introduced as a sodium salt. The electrophysiological recording procedure was the same as that described in Hao et al. (2016). The ramping voltage program used to capture currents is shown in Supplementary Figure 1A. Briefly, the oocytes were clamped to −70 mV, with the exception of a voltage ramp (increased from −160 to +20 mV in 1.5 s) applied every 70 s. The obtained current-voltage relationship curves in the presence or absence of ammonium are illustrated in Supplementary Figures 1B–D. The ammonium-induced current was calculated using the following formula:

Ammonium-induced⁢current⁢(I)=Current-containingammoniumCurrent.before⁢ammonium⁢addition

The methylammonium-induced currents were calculated in a similar way, by subtraction of the background currents obtained at methylammonium-free solution from the total currents in the presence of methylammonium. Similar to previous reports (Ludewig et al., 2002; Yang et al., 2015), oocytes injected with H_2_O (control oocytes) did not produce significant endogenous currents, under conditions of ≤1 mM ammonium or ≤10 mM methylammonium in bath solution. The animal study was reviewed and approved by Laboratory Animal Resources, Chinese Academy of Sciences. Accordingly, *Xenopus laevis* handling was performed according to its standard biosecurity and institutional safety procedures.

### Sequence Alignments and Data Analyses

The cytosolic carboxyl-terminus region sequence alignments of AMTs were performed using DNAMAN 6.0 software. The maize growth and ammonium uptake experiments were both repeated three times. Each data point in the electrophysiological test was derived from at least 3 oocytes from different batches. Statistical analysis was performed using SPSS 20.0 software. Graphs were generated using Sigmaplot 12.5 software.

## Results

### Ammonium Transport Kinetics of Three ZmAMT1s in Oocytes

**FIGURE 1:**
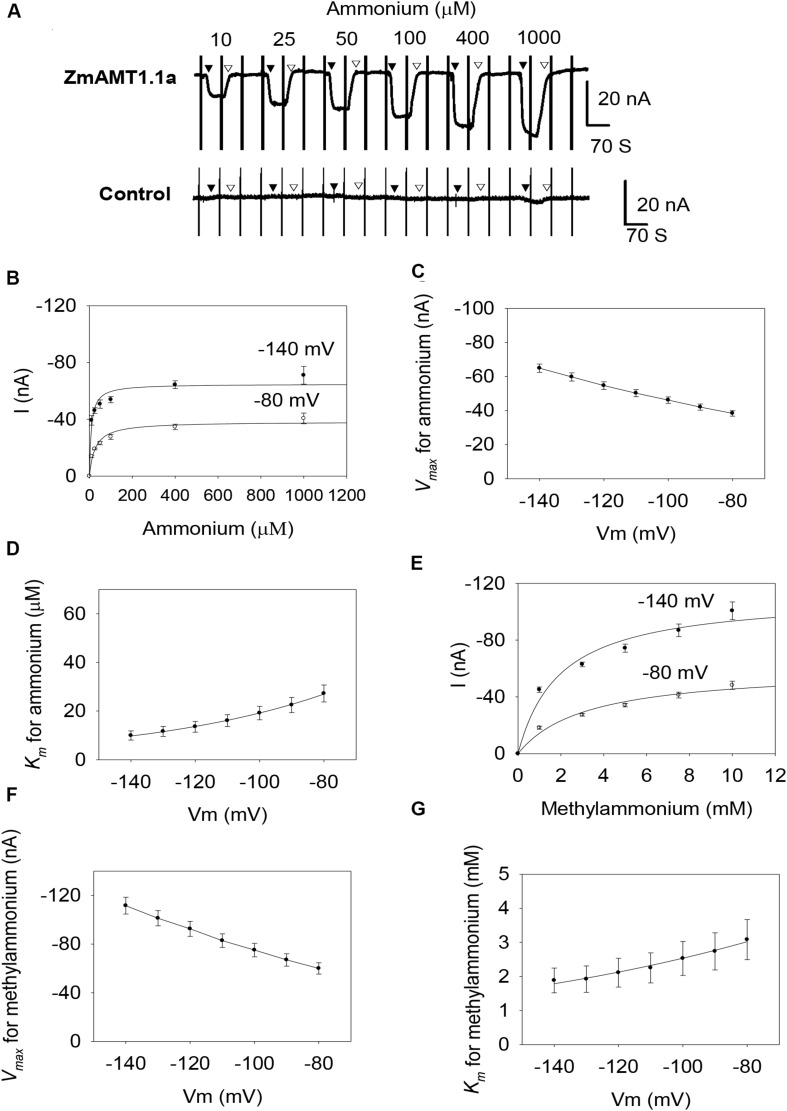
Transport kinetics of ZmAMT1.1a for ammonium and its analog methylammonium in *Xenopus* oocytes. **(A)** Representative recordings showing the current responses of oocytes injected with ZmAMT1.1a (upper panel) or an equal volume of water (control, lower panel) to different perfused ammonium concentrations. The oocytes were subjected to ammonium-free solution, followed by an ammonium solution, a return to ammonium-free solution, and then the next ammonium solution until all solutions had been used. The introduced ammonium (added as a chloride salt) concentrations in the bath solutions were 10, 25, 50, 100, 400, and 1000 μM, in that order. The oocytes were clamped at –70 mV, with the exception of a voltage ramp (from –160 to +20 mV in 1.5 s) applied every 70 s. The introduction (▼) or withdrawal (▽) of the ammonium is indicated. **(B)** Kinetics of ammonium transport for ZmAMT1.1a at membrane potentials of –80 or –140 mV. The inserted 1.5 S ramp program, as illustrated in A, captured the corresponding currents response at different membrane potentials, either in the presence or absence of ammonium. Ammonium induced current (I) = Current _*containing*__*ammonium*_–Current _*before ammonium addition*_. Curves represent the fitted Michaelis-Menten equations that describe the relationship between the supplied ammonium concentration and the corresponding ammonium-induced current. **(C)** Maximum ammonium absorption rate *V*_*max*_ for ZmAMT1.1a at membrane potentials ranging from –80 to –140 mV. **(D)** Voltage dependence of *K*_*m*_ values for ammonium. The curve represents the fitted exponential equation. **(E)** Kinetics of methylammonium transport for ZmAMT1.1a at –80 or –140 mV. Oocytes were subjected to the same procedure as the ammonium perfusion in A, with the exception of substitution of the ammonium with methylammonium. The methylammonium (added as a chloride salt) concentrations applied were 1, 3, 5, 7.5, and 10 mM in order. The methylammonium-induced current (I) was obtained by subtracting the “background” current in the absence of methylammonium from the total current in the presence of methylammonium. **(F)** Maximum methylammonium absorption rate *V*_*max*_ for ZmAMT1.1a at membrane potentials ranging from –80 to –140 mV. **(G)** Voltage dependence of *K*_*m*_ values for methylammonium. The curve represents a fitted exponential equation describing the relationship between the membrane potentials and the corresponding *K*_*m*_ values; *n* = 3. The data shown are the mean ± SE.

Screening of maize root cDNA library using yeast mutant found that there were only three functional AMTs in maize roots, being ZmAMT1.1a, ZmAMT1.1b and ZmAMT1.3 (Gu et al., 2013). However, their functional properties, especially their functional modulations, remain largely unclear. With these questions, the three ZmAMT1s were firstly heterologously expressed in oocytes, and then were investigated by the two-electrode voltage clamp technique. The steady-state background currents were first recorded following the exposure of the ZmAMT1.1a-expressing oocytes to the ammonium-free bath solution (Figure 1A). Upon the introduction of ammonium (e.g., 10 μM) into the ammonium-free solution, the ammonium-induced currents in ZmAMT1.1a immediately appeared and rapidly reached a plateau. Subsequent withdrawal of ammonium from the bath solution led to a return of the currents back to the level of previous background currents. Increasing the externally supplied ammonium concentration (ranging from 10 to 1000 μM) led to ammonium-induced currents of increasing magnitude (Figure 1A). In control oocytes, when ammonium was introduced into the bath solution using the same procedure as that for ZmAMT1.1a, no ammonium-activated currents were induced, regardless of the supplied ammonium concentrations (Figure 1A). This indicates that under ≤1 mM ammonium, the control oocytes do not produce an endogenous current, which is consistent with previous reports (Ludewig et al., 2002; Yang et al., 2015). The occurrence of the ammonium-induced currents in ZmAMT1.1a, together with the absence of ammonium-activated currents in control oocytes, directly supported the idea that ZmAMT1.1a could mediate the electrogenic influx of ammonium. The transport activity of ZmAMT1.1a increased with increasing concentration of applied ammonium and reached saturation at approximately 1000 μM, for both −140 and −80 mV (Figure 1B). The relationship between the ammonium concentration and the corresponding ammonium-induced currents could be described by the Michaelis-Menten equation (Figure 1B). In the membrane potential range of −140 to −80 mV, the maximum ammonium absorption rate (*V*_*max*_) was voltage-dependent, with the *V*_*max*_ values increasing as with more negative membrane potentials, ranging from −38.3 ± 1.6 to −64.9 ± 2.5 nA (Figure 1C). The ammonium concentration (*K*_*m*_) needed to achieve half the maximum absorption rate was also voltage-dependent, but operated in an opposite manner to that of *V*_*max*_. The *K*_*m*_ values decreased with increasing negative membrane potentials, ranging from 9.9 ± 1.9 to 27.2 ± 3.5 μM (Figure 1D). These data suggest that ZmAMT1.1a is a voltage- and concentration-dependent high-affinity ammonium transporter. The Hill coefficient derived from the fitted Michaelis-Menten equation was equal to 1 (Figure 1B), suggesting that only one type of substrate binding event occurs in ZmAMT1.1a. The more negative the membrane potential, the lower the *K*_*m*_ value (Figure 1D), suggesting that the substrate bound to the binding site is a cation. Investigation of methylammonium, an ammonium analog, led to similar voltage- and concentration-dependent transport properties: increasing methylammonium-induced current with increasing methylammonium concentration, larger *V*_*max*_, and smaller *K*_*m*_ values at more negative membrane potentials (Figures 1E–G). In the membrane potential range of −140 to −80 mV, the *K*_*m*_ for methylammonium increased from 1.9 ± 0.4 mM to 3.1 ± 0.6 mM (Figure 1G), suggesting that ZmAMT1.1a mediates low-affinity transport of methylammonium.

**FIGURE 2:**
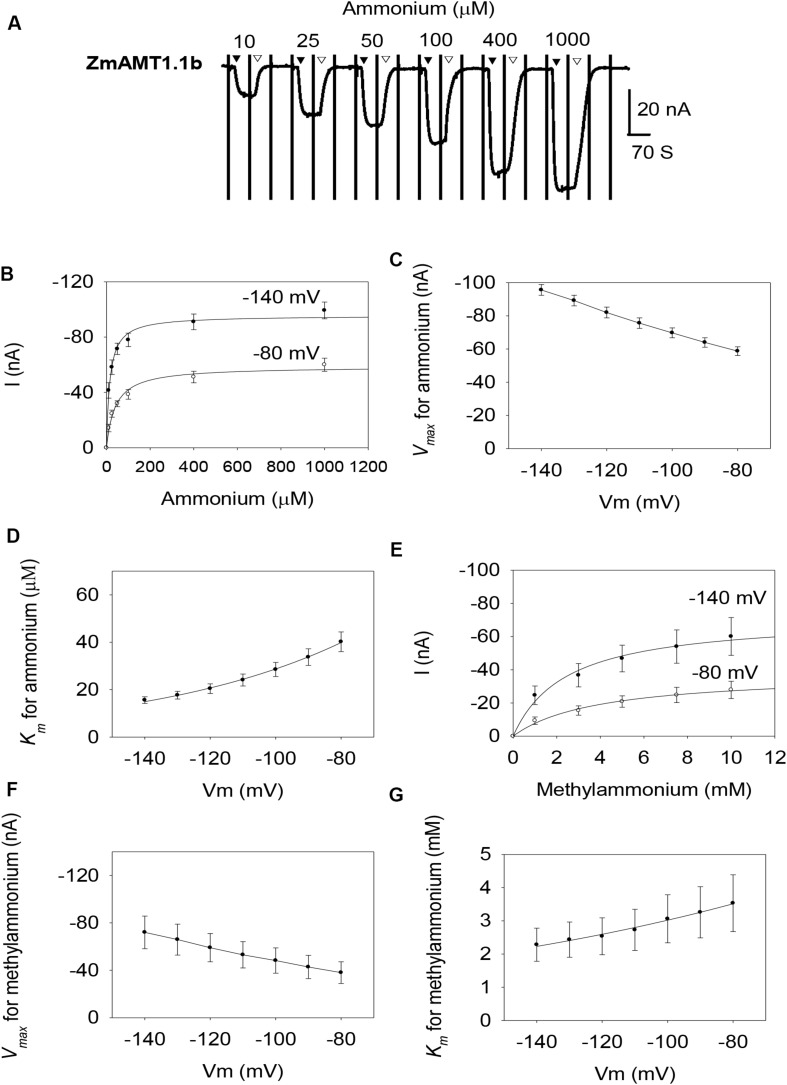
Ammonium and methylammonium transport kinetics of ZmAMT1.1b in oocytes. **(A)** Representative recordings showing the current responses of ZmAMT1.1b-expressing oocytes to different perfused ammonium concentrations. Ammonium was added as a chloride salt. The protocol used is described in Figure 1, and the introduction (▼) or withdrawal (▽) of ammonium is indicated. **(B)** Ammonium transport kinetics for ZmAMT1.1b at –80 or –140 mV. Curves represent the fitted Michaelis-Menten equation. **(C)** Maximum ammonium absorption rate *V*_*max*_ for ZmAMT1.1b at membrane potentials ranging from –80 to –140 mV. **(D)** Voltage dependence of *K*_*m*_ values for ammonium of ZmAMT1.1b. The curve represents the fitted exponential equation. **(E)** Kinetics of methylammonium transport for ZmAMT1.1b. Methylammonium was added as a chloride salt. **(F)** Maximum methylammonium absorption rate *V*_*max*_ for ZmAMT1.1b at membrane potentials ranging from –80 to –140 mV. **(G)** Voltage dependence of *K*_*m*_ values for methylammonium in ZmAMT1.1b. The curve represents the fitted exponential equation. *n* = 3 for panels **(B–D)** and *n* = 5 for panels **(E–G)**. The data shown are the mean ± SE.

**FIGURE 3:**
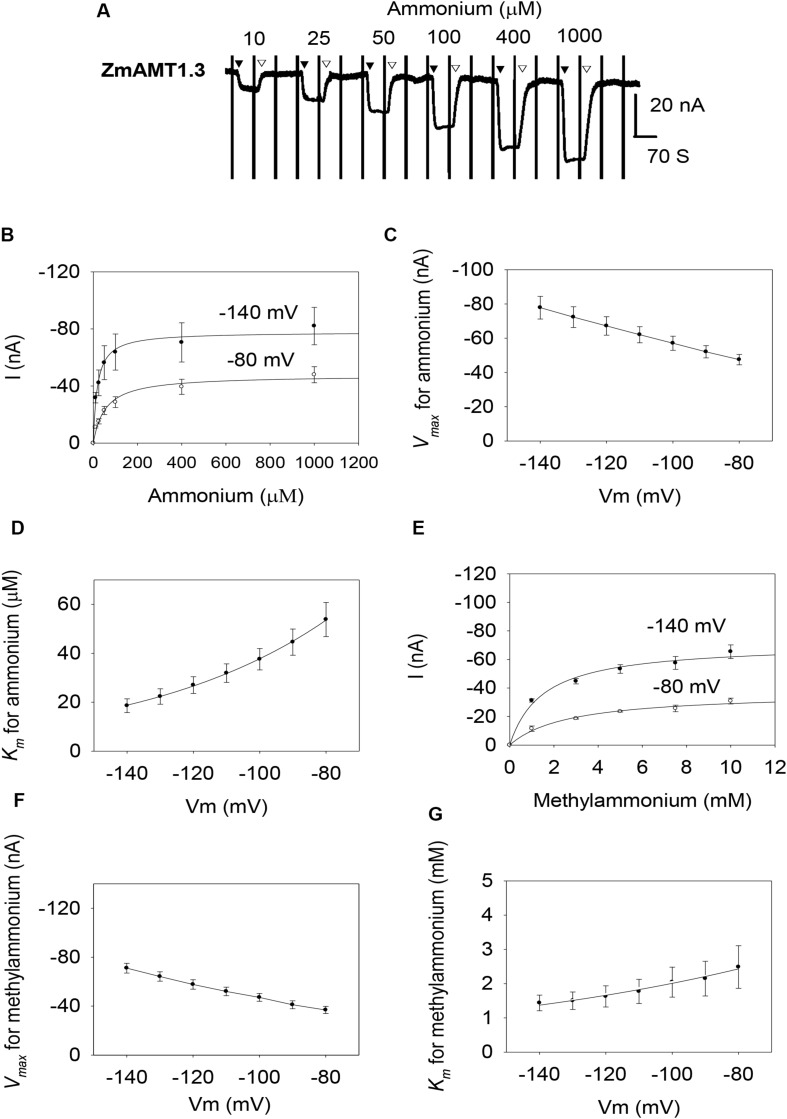
Ammonium and methylammonium transport kinetics of ZmAMT1.3 in oocytes. **(A)** Representative recordings showing the current responses of ZmAMT1.3-expressing oocytes to various perfused ammonium concentrations. Ammonium was added as a chloride salt and the protocol used is described in Figure 1. The introduction (▼) or withdrawal (▽) of ammonium is indicated. **(B)** Kinetics of ammonium transport by ZmAMT1.3 at –80 or –140 mV. Curves represent the fitted Michaelis-Menten equations. **(C)** Maximum ammonium absorption rate *V*_*max*_ for ZmAMT1.3 at membrane potentials ranging from –80 to –140 mV. **(D)** Voltage dependence of *K*_*m*_ values for ammonium in ZmAMT1.3. The curve represents the fitted exponential equation. **(E)** Kinetics of methylammonium transport for ZmAMT1.3. Methylammonium was added as a chloride salt. **(F)** Maximum methylammonium absorption rate *V*_*max*_ for ZmAMT1.3 at membrane potentials ranging from –80 to –140 mV. **(G)** Voltage dependence of *K*_*m*_ values for methylammonium in ZmAMT1.3. The curve represents the fitted exponential equation, *n* = 3. The data shown are the mean ± SE.

Similar to ZmAMT1.1a, both ZmAMT1.1b and ZmAMT1.3 are high-affinity ammonium transporters with ∼100-fold lower affinity to methylammonium (Figures 2, 3). Based on the Hill coefficients obtained from the fitted Michaelis-Menten equations (Figures 2B,E, 3B,E), together with the voltage dependences of the ammonium and methylammonium *K*_*m*_ values (Figures 2D,G, 3D,G), we propose that both ZmAMT1.1b and ZmAMT1.3 transport their substrate in the cationic form, similar to that of ZmAMT1.1a.

However, the three ZmAMT1s still exhibited differences in their ammonium kinetics. In the membrane potential range of −140 to −80 mV, both ZmAMT1.1b and ZmAMT1.3 showed higher *V*_*max*_ (∼50% and ∼20%, respectively) and *K*_*m*_ (∼50% and ∼90%, respectively) values for ammonium, than ZmAMT1.1a (Figures 1C,D, 2C,D, 3C,D). With regard to the methylammonium kinetics, ZmAMT1.1a showed the largest *V*_*max*_ values for methylammonium (Figures 1F, 2F, 3F), and ZmAMT1.1b showed the largest *K*_*m*_ values for methylammonium, among the three ZmAMT1s (Figures 1G, 2G, 3G). We noticed here that ZmAMT1.1a showed the lowest ammonium transport activity, but with the highest capability of methylammonium transport among the three ZmAMT1s. This unusual phenomenon is similar to the Q57H mutation in AtAMT1;1 (Loqué et al., 2009) and the H211E mutation in PvAMT1;1 (Ortiz-Ramirez et al., 2011), both of which led to enhanced transport of ammonium, but reduced transport of methylammonium. Therefore, caution is required when using methylammonium absorption to represent the transport activity of AMTs (Ortiz-Ramirez et al., 2011).

### Effect of Extracellular pH on the Transport Activity of ZmAMT1s

**FIGURE 4:**
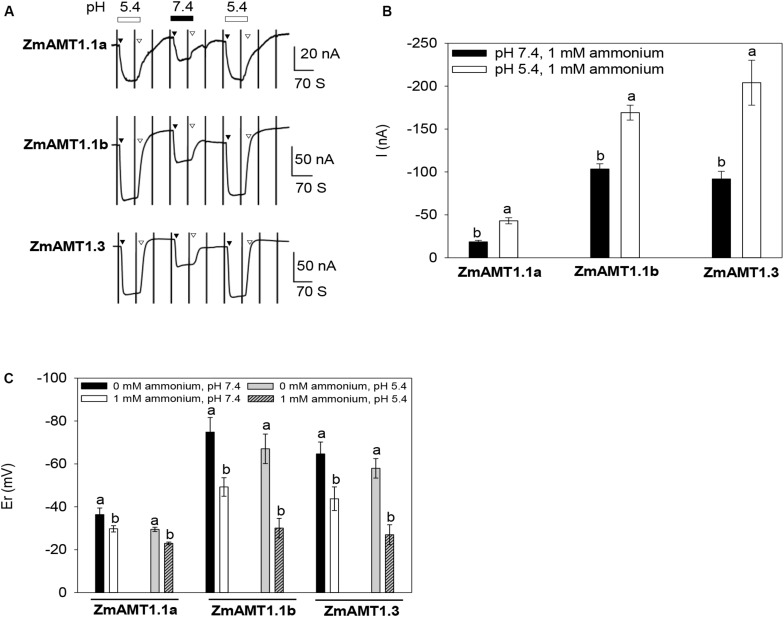
Effect of extracellular pH on the transport activity of ZmAMT1s. **(A)** Representative recordings showing the current responses of ZmAMT1s to 1 mM ammonium at different pH values. The protocol used is described in Figure 1. The introduction (▼) or withdrawal (▽) of 1 mM ammonium at different pH values is indicated. Ammonium was added as a chloride salt. **(B)** Amplitudes of the induced currents in ZmAMT1s in the presence of 1 mM ammonium at different pH values (–140 mV). **(C)** Reversal potential (Er) of ZmAMT1 in the presence or absence of 1 mM ammonium at different pH values. Er, the membrane potential that corresponds to where the current equals zero. In panels **(B,C)**, *n* = 3, 3 for ZmAMT1.1a, *n* = 4, 6 for ZmAMT1.1b, and *n* = 3, 6 for ZmAMT1.3. Different letters represent significant differences between adjacent treatments (Student’s *t*-test, *P* < 0.05). The data shown are the mean ± SE.

Acidification of the growth medium occurs during ammonium absorption in plants (Zhu et al., 2009). Two electrode voltage clamp recording was done with bath solutions at different pH values (5.4 vs. 7.4) to investigate the effect of medium acidification on the transport activity of ZmAMT1s. Since ammonium ion is the major component at these two pH values, differences in transport are likely to represent proton-coupled transport rather than small differences in ammonium ion concentrations. The results showed that the alkalinization of (from 5.4 to 7.4) the bath solution significantly inhibited the ammonium transport activity of the three ZmAMT1s, while acidification of the bath solution (from 7.4 to 5.4) sharply enhanced their transport activity (Figure 4A). This demonstrates that each ZmAMT1 is sensitive to external pH and is able to quickly change its ammonium transport activity to accommodate the changing pH value of the medium. The two-unit decrease of the pH value (5.4 vs. 7.4) increased the transport activity by 140.8%, 64.1% and 120.7%, respectively, for ZmAMT1.1a, ZmAMT1.1b and ZmAMT1.3 (Figure 4B). The reversal potential (Er) of the three ZmAMT1s moved toward the positive direction after ammonium introduction, regardless of the pH value (Figure 4C), suggesting that each ZmAMT1 is permeable to ammonium. When the pH was 5.4, the magnitude of change in Er after ammonium addition (△Er, △Er = Er _1ammonium_ -Er _0ammonium_) was slightly higher than that when the pH was 7.4, but the difference was not statistically significant. This indicates that protons are not substrate transported by ZmAMT1s. Together with the conclusions drawn from Figures 1–3, the data support the conclusion that the cationic substrate transported by ZmAMT1s is NH_4_^+1^. This type of ionic NH_4_^+^ uniport transport mechanism has been reported for several plant AMTs (Ludewig et al., 2002; Wood et al., 2006; Loqué et al., 2009; Yang et al., 2015).

### Effects of Exogenous Nitrate, Gln, and Ca^2+^ on the Transport Activity of ZmAMT1s

**FIGURE 5:**
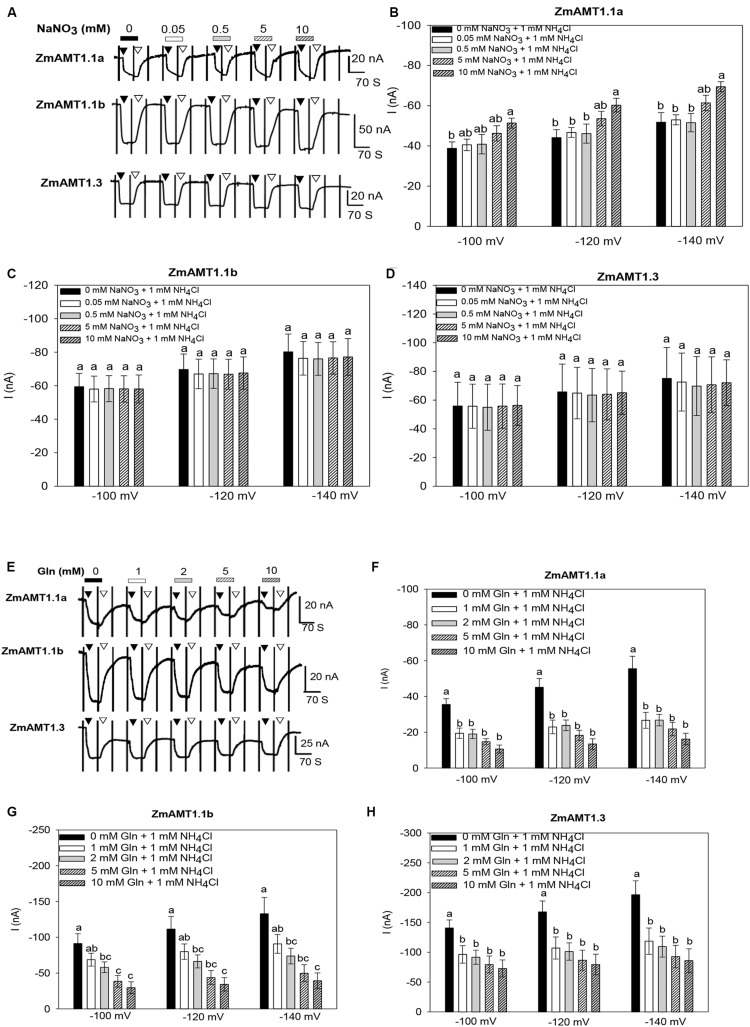
Effect of the exogenous introduction of nitrate, Gln or Ca^2+^ on the transport activity of ZmAMT1s. **(A)** Representative recordings showing the current responses of ZmAMT1s-expressing oocytes to 1 mM ammonium in the co-existence of different concentrations of nitrate. Ammonium was added in the form of NH_4_Cl. Nitrate was added in the form of NaNO_3_. The oocytes were sequentially subjected to changing bath solutions as indicated. The protocol used is described in Figure 1. The introduction (▼) or withdrawal (▽) of 1 mM ammonium in the presence of different concentrations of nitrate is indicated. **(B–D)** Currents induced by 1 mM ammonium in the presence or absence of different concentrations of nitrate for ZmAMT1.1a **(B)**, ZmAMT1.1b **(C)**, and ZmAMT1.3 **(D)** at –100, –120, and –140 mV. **(E)** Representative recordings showing the current responses of ZmAMT1s-expressing oocytes to perfusion with 1 mM ammonium in the co-existence of different concentrations of Gln. **(F–H)** Currents induced by 1 mM ammonium in the presence or absence of different concentrations of Gln for ZmAMT1.1a **(F)**, ZmAMT1.1b **(G)**, and ZmAMT1.3 **(H)** at –100, –120, and –140 mV. Different letters represent significant differences among treatments (LSD, *P* < 0.05). In panels **(B–D)**, *n* = 3, 5, 5. In panels **(F–H)**, *n* = 5, 6, 3. The data shown are the mean ± SE.

Maize grows in soils where ammonium and nitrate co-exist. We added different concentrations of nitrate to the bath solution to simulate the condition of nitrate co-existence with ammonium. Under the condition of fixed 1 mM ammonium exposure, the addition of different concentrations of nitrate (ranged from 0.05 to 10 mM) had no significant effect on ZmAMT1.1b- or ZmAMT1.3-mediated ammonium-induced currents under different membrane potentials (Figures 5A,C,D), indicating that under our experimental conditions, the transport activities of these two ZmAMT1s were not regulated by nitrate. With respect to ZmAMT1.1a, although its transport activity was unchanged in the presence of low concentrations of nitrate (from 0.05 to 5 mM), a significantly enhanced transport activity occurred in the presence of 10 mM nitrate. Both cases showed similar trends at −100, −120, and −140 mV (Figures 5A,B).

Glutamine (Gln) is an important product of plant ammonium assimilation (Hodges, 2002). When the ammonium concentration was fixed to 1 mM, exogenous addition of 1 mM Gln significantly inhibited the ammonium transport activity of ZmAMT1.1a and ZmAMT1.3. Further increases in the concentration of Gln did not result in additional inhibition of these two proteins ammonium currents (Figures 5E,F,H). In contrast, Gln inhibition of ZmAMT1.1b was concentration-dependent (Figures 5E,G). At −140 mV, addition of 10 mM Gln suppressed the transport activity of ZmAMT1.1a, ZmAMT1.1b, and ZmAMT1.3 by 69.9%, 67.5%, and 55.4%, respectively (Figures 5F–H).

It has been reported that changes in the extracellular Ca^2+^ concentration can regulate the function of ion channels (Leng et al., 2002). To verify whether there was a similar Ca^2+^ regulation pattern in transporters such as ZmAMT1s, Ca^2+^ concentration assays were conducted. We found that either a 10-fold reduction in the Ca^2+^ concentration of the bath solution (from 2 to 0.2 mM), or a 5-fold increase in the Ca^2+^ concentration (from 2 to 10 mM) did not have a significant impact on the transport activity of the three ZmAMT1s (Supplementary Figure 2), indicating they are not sensitive to extracellular Ca^2+^ concentration.

### Effect of Mutation of a Conserved Threonine Located at the Carboxyl-Terminus on the Transport Activity of ZmAMT1s

**FIGURE 6:**
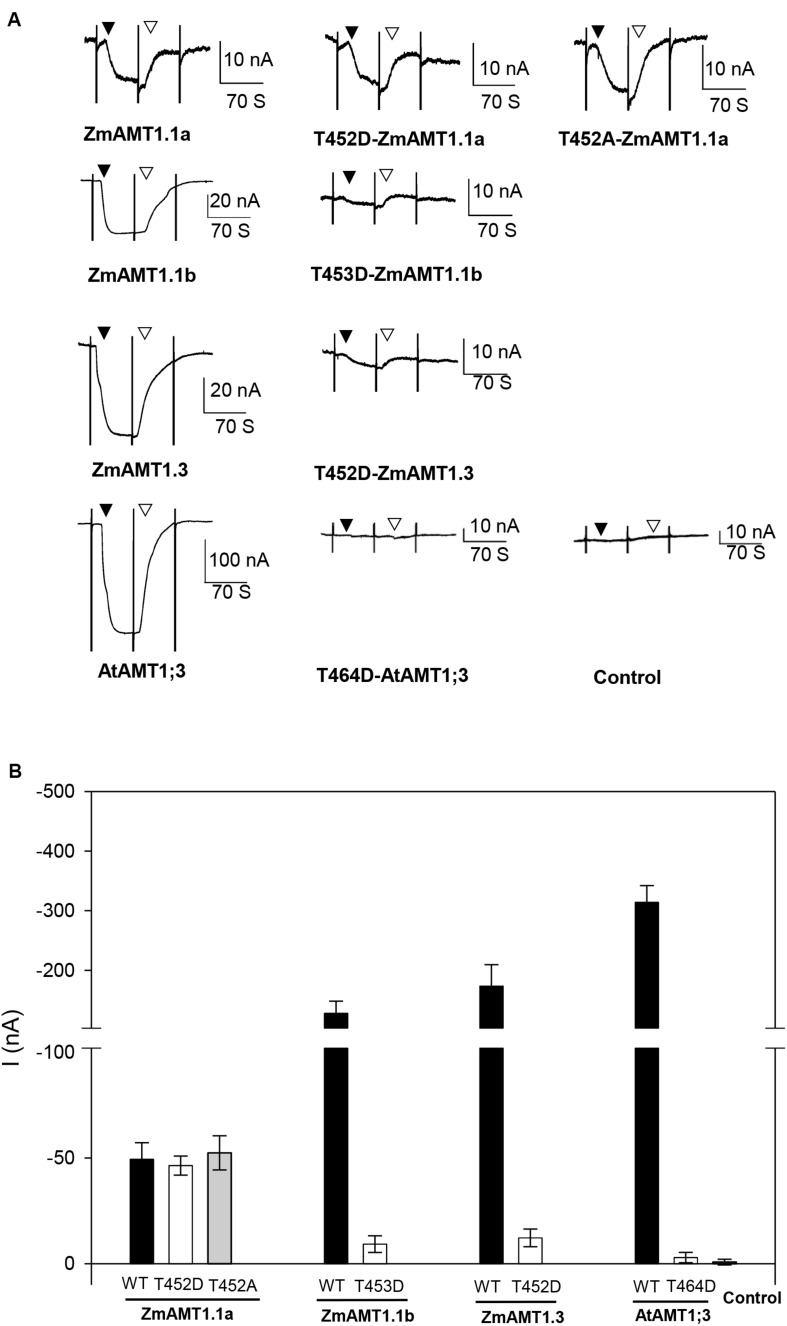
Effect of the carboxyl-terminus conserved threonine mutation on the transport activity of ZmAMT1s. **(A)** Representative recordings showing the currents responses of ZmAMT1s, AtAMT1;3 wild type, and their “T460” corresponding mutants to 1 mM ammonium. The “T464” site in AtAMT1;3, the “T452” site in ZmAMT1.1a, the “T453” site in ZmAMT1.1b, and the “T452” site in ZmAMT1.3 correspond to the T460 site in AtAMT1;1. The reported T464D-AtAMT1;3 (Yuan et al., 2013) was used as a technical control. The introduction (▼) or withdrawal (▽) of ammonium (added as a chloride salt) is indicated. **(B)** Ammonium-induced currents in wild-type AMT1s and their “T460” corresponding site mutants to 1 mM ammonium. *n* = 5, 5, 6, 3, 4, 3, 3, 4, 3, and 3 for ZmAMT1.1a, T452D-ZmAMT1.1a, T452A-ZmAMT1.1a, ZmAMT1.1b, T453D-ZmAMT1.1b, ZmAMT1.3, T452D-ZmAMT1.3, AtAMT1.3, T464D-AtAMT1.3, and the control, respectively. The data shown are the mean ± SE.

Studies have shown that the transport activity of plant AMTs is regulated by the phosphorylation status of a highly conserved threonine (“T460”) located at its carboxyl-terminus. Substitution of this amino acid with glutamate (D) led to essential loss-of-function of several plant AMTs (Loqué et al., 2007; Neuhäuser et al., 2007; Yuan et al., 2013; Guo et al., 2018; Wu et al., 2019). Sequence alignment among plant AMTs showed that each ZmAMT1 contained a corresponding threonine residue (Supplementary Figure 3). To investigate whether such a substitution at this corresponding site in ZmAMT1 produced a similar effect as that in AtAMT1;1, we firstly constructed the “T” to “D” mutants of ZmAMT1s, respectively, named T452D-ZmAMT1.1a, T453D-ZmAMT1.1b, and T452D-ZmAMT1.3, and then expressed these mutants in oocytes to monitor their effects on ZmAMT1s’ transport activity. A previously reported mutant T464D at the corresponding site of AtAMT1;3 was used as a technical control. Significant ammonium-induced currents were detected in AtAMT1;3-expressing oocytes, but were not observed in either of the T464D-AtAMT1;3-expressing and control oocytes (Figures 6A,B), suggesting that AtAMT1;3 is a functional ammonium transporter and T464D-AtAMT1;3 is a loss-of-function mutant, which is in accordance with a previous report (Yuan et al., 2013). With respect to ZmAMT1s, the T453D and T452D mutations, respectively, led to a 92.8% and 93% reduction in the ammonium-induced currents of their wild types, ZmAMT1.1b, and ZmAMT1.3 (Figures 6A,B). The currents activated by ammonium in T453D-ZmAMT1.1b and T452D-ZmAMT1.3 were indistinguishable from the currents induced by ammonium in T464D-AtAMT1;3 and the control (Figures 6A,B). These data suggest that mutations of T453D and T452D essentially cause loss-of-function of ZmAMT1.1b and ZmAMT1.3. The corresponding position mutation of T452D in ZmAMT1.1a did not affect its transport activity, as evidenced by the comparable ammonium-activated currents between the T452D-ZmAMT1.1a and the wild type ZmAMT1.1a. Similarly, another type of mutation at this site, T452A, also did not affect the transport activity of ZmAMT1.1a (Figures 6A,B). These results suggest that the T452 residue in ZmAMT1.1a may act differently from its equivalent residues in the other two ZmAMT1s.

### Ammonium Uptake and Regulation in Maize Roots

**FIGURE 7:**
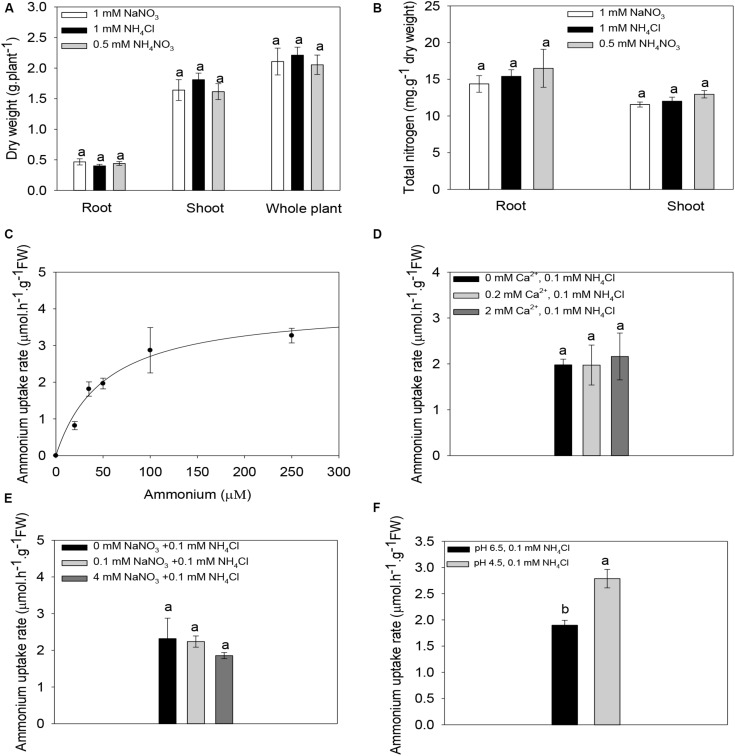
Ammonium absorption properties of maize roots. **(A,B)** Biomass **(A)** and nitrogen content **(B)** of maize seedlings under different nitrogen forms. Maize seedlings were pre-cultured for 14 days in nutrient solution containing 0.5 mM NH_4_NO_3_. Uniform seedlings then received treatment with nitrate only (1 mM nitrate), ammonium only (1 mM ammonium) or ammonium nitrate (0.5 mM NH_4_NO_3_) for 10 days for the biomass and nitrogen content determination. **(C)** Kinetics of ammonium absorption in maize roots. The curve represents the fitted Michaelis-Menten equation that describes the relationship between the supplied ammonium concentration and the ammonium uptake rate. **(D–F)** The Effect of the exogenous addition of Ca^2+^ **(D)**, or nitrate **(E)** or changes in the pH value of the ammonium uptake solution **(F)** on the ammonium absorption rate in maize roots. Maize seedlings were pre-cultured for 14 days in nutrient solution containing 0.5 mM NH_4_NO_3_ and then subjected to a nitrogen-free treatment for 3 days. The roots were finally immersed in a ammonium uptake solution containing different concentrations of ammonium **(C)**, 0.1 mM ammonium in the presence of different concentrations of Ca^2+^ **(D)**, 0.1 mM ammonium in the presence of different concentrations of nitrate **(E)**, or 0.1 mM ammonium at different pH values **(F)**, for ammonium uptake rate determinations. *n* = 14 for panel **(A)**, *n* = 8 for panel **(B)**, and *n* = 3 for panels **(C–F)**. Different letters represent significant differences among treatments [LSD for panels **(A–E)**; Student’s *t*-test for panel **(F)**, *P* < 0.05]. The data shown are the mean ± SE.

To establish a link between the functional properties of ZmAMT1s and their possible physiological significance *in planta*, we carried out hydroponic experiments using maize seedlings as materials. When subjected to either ammonium only (1 mM NH_4_Cl), nitrate only (1 mM NaNO_3_), or ammonium nitrate (0.5 mM NH_4_NO_3_) treatment for 10 days, there were no differences in root, shoot, or whole-plant dry weight (Figure 7A), or in root and shoot nitrogen content (Figure 7B) among treatments. This indicates that although maize grows in nitrate-dominated dryland soils, ammonium nitrogen has a growth-supporting effect comparable to that of nitrate nitrogen alone, at least under hydroponic conditions. The ammonium uptake rate in maize roots increased with increasing concentration of the externally supplied ammonium and reached saturation at approximately 250 μM (Figure 7C). The ammonium absorption kinetics conformed to the Michaelis-Menten equation. The *V*_*max*_ value of maize roots was 4.1 ± 0.6 μmol.h^–1^.g^–1^ FW, and the *K*_*m*_ value was 51.8 ± 17.9 μM. This demonstrates that the maize roots possess a high-affinity ammonium absorption system. Under the condition of fixed 0.1 mM ammonium exposure, changes in the Ca^2+^ concentration in the ammonium uptake solution had no significant effect on the ammonium absorption of maize roots (Figure 7D). Similarly, the exogenous addition of different concentrations of nitrate did not significantly affect the ammonium uptake of maize roots (Figure 7E). Notably, a two-unit decrease in the pH value (pH 4.5 vs. pH 6.5) of the ammonium uptake solution significantly increased the ammonium absorption rate by 48.2% (Figure 7F).

### Gene Expression Profiles of ZmAMT1s Under Different Forms of Nitrogen Treatments

**FIGURE 8:**
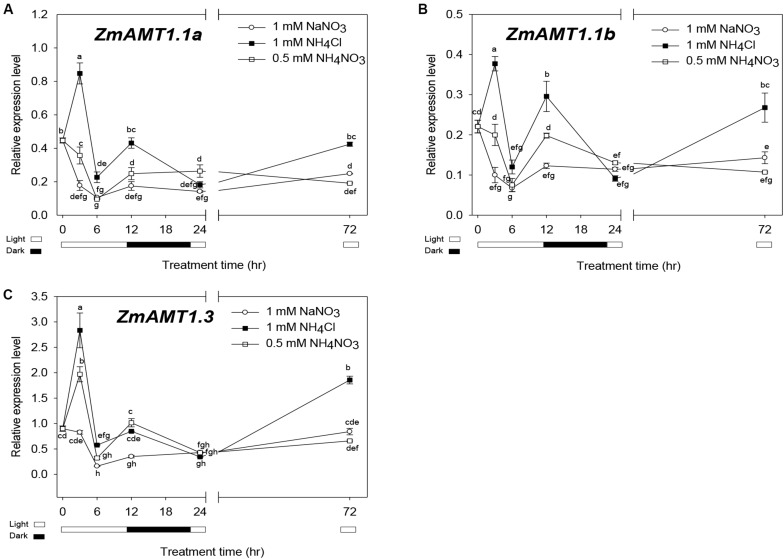
Expression patterns of *ZmAMT1s* under different forms of nitrogen treatments in maize roots. **(A–C)** The transcript abundances of *ZmAMT1.1a* **(A)**, *ZmAMT1.1b* **(B)**, and *ZmAMT1.3* **(C)** in maize roots under the different forms of nitrogen treatments. Maize seedlings were pre-cultured for 14 days in nutrient solution containing 0.5 mM NH_4_NO_3_. Uniform seedlings then received the treatment with nitrate only (1 mM NaNO_3_), ammonium only (1 mM NH_4_Cl) or ammonium nitrate (0.5 mM NH_4_NO_3_) for 0, 3, 6, 12, 24, and 72 h, for real-time quantitative PCR analysis. The light condition (□, light and ■, dark) is indicated. Expression values were calculated using the 2^–Δ^ ^CT^ method with *ZmACT1* (J01238) as an endogenous control. Different letters represent significant differences among treatments (LSD, *P* < 0.05); *n* = 3. The data shown are the mean ± SE.

Figures 8A–C shows time-course expression profiles of *ZmAMT1.1a*, *ZmAMT1.1b*, and *ZmAMT1.3* in response to different conditions of nitrogen forms. One mM NaNO_3_ was set as a “normal control” to simulate nitrate-dominated conditions. Compared to this control, the transcript abundances of all three genes were rapidly up-regulated in roots in the presence of ammonium (0.5 mM NH_4_NO_3_) within 3 h, and peaked in the ammonium-dominated environment (1 mM NH_4_Cl). This transcriptional stimulation may correspond to a prompt enhancement of ammonium uptake activity when this form of nitrogen source is available to maize roots. After peaking at 3 h, the transcript levels declined and oscillated along extended time course (Figures 8A–C).

We also noticed that the oscillating changes of the transcript abundances of *ZmAMT1.1a*, *ZmAMT1.3* and *ZmAMT1.1b* in the presence of ammonium over the 6 to 24 h time-course corresponded to a shift in light/dark cycles (Figures 8A–C). Lower abundances were observed in the first light cycle (6 h), followed by higher levels in the dark (12 h) and a return to lower abundances with the restoration of the light cycle (24 h). This might be, an implication that the expression of the three *AMT* genes in maize roots is also regulated by the circadian rhythm, a phenomenon that was previously reported for *LeAMT1;2/LeAMT1;3* (Von Wirén et al., 2000), *PtrAMT1;6* (Couturier et al., 2007), and *OsAMT1;1* (Ranathunge et al., 2014).

## Discussion

### ZmAMT1 Proteins Show Differential Ammonium Absorption Kinetics

The expression of the three ZmAMT1 genes in *Xenopus* oocytes mediated the electrogenic influx of ammonium (Figures 1A, 2A, 3A), and their affinity constant *K*_*m*_ values for ammonium varied from 9.9 ± 1.9 to 18.6 ± 2.8 μM (Figures 1D, 2D, 3D), indicating that all three ZmAMT1s are functional high-affinity ammonium transporters. Compared with *K*_*m*_ values from other plant AMTs using the same research technique (7.3−140 μM; Ludewig et al., 2002; Wood et al., 2006; Neuhäuser et al., 2007; Loqué et al., 2009; Ortiz-Ramirez et al., 2011; Yang et al., 2015; Hao et al., 2016; Guo et al., 2018; Wu et al., 2019), the affinities of these three ZmAMT1s are in a relatively higher affinity range. The concentration of ammonium in soils is lower than 1 mM, and sometimes rarely exceeds 50 μM (Yuan et al., 2007). It is thus speculated that the relatively high affinities of ZmAMT1s might be more conducive to the efficient absorption of ammonium in dryland soils. Despite the fact that they are all high-affinity ammonium transporters, there was a significant difference in ammonium affinity and maximum absorption rate among the three ZmAMT1s (Figures 1C,D, 2C,D, 3C,D). This is similar to the three major AtAMT1s in Arabidopsis (Yuan et al., 2007). Given that this variation in affinity in Arabidopsis is required for high-efficiency ammonium uptake, it is speculated that these three ZmAMT1s may play similar physiological roles in maize.

### Maize May Achieve Efficient Ammonium Acquisition by Virtue of Acidification Caused by an Ammonium-Dominated Environment

The electrophysiological results showed that the transport activities of all three ZmAMT1s were strongly promoted by acidification of the medium (Figure 4). Among the three LeAMT1s in tomato, the functionality of LeAMT1;1 and LeAMT1;2 is resistant to external pH changes (Ludewig et al., 2002; Ludewig et al., 2003). Among the five AtAMT1s in Arabidopsis, the function of AtAMT1;1 is insensitive to external pH (Wood et al., 2006; Loqué et al., 2009). Among the three OsAMT1s in rice (Gaur et al., 2012), the function of OsAMT1;1 is unchanged against external pH changes (Yang et al., 2015). The function of TaAMT1;1 in wheat and PvAMT1;1 in common bean is promoted by low pH (Søgaard et al., 2009; Ortiz-Ramirez et al., 2011), but the pH regulatory mode in other TaAMTs or PvAMTs is unknown (Li et al., 2017). Unlike these other plant systems, we find that ZmAMT1 is currently known the only group among plant AMTs in which the activity of all its members is promoted by low pH. The acid-stimulated transport activities of ZmAMT1s (Figures 4A,B) were consistent with the observation of acid-facilitated ammonium absorption capacity of maize roots (Figure 7F). In agricultural fields, nitrogen topdressing (such as during the jointing stage and the large flare stage) can create ammonium-dominated conditions in a short time in the maize-growing soil environment, which is accompanied by rhizosphere acidification (Zhu et al., 2009). The strongly acid-stimulated absorption of ammonium might be a strategy used by maize roots to efficiently obtain ammonium nutrients through ZmAMT1s. Additionally, at the transcriptional level, introduction of ammonium to maize roots sharply up-regulated the gene expression abundances of *ZmAMT1s* compared to the transcript abundances in the nitrate-dominated environment, and their transcript abundances were further enhanced by increases in ammonium proportion in solutions (Figure 8). This transcriptional stimulation might also help maize to achieve efficient absorption of ammonium nitrogen after nitrogen fertilizer application (a case of ammonium domination in soils).

The independence of ZmAMT1s’ transport activity on extracellular Ca^2+^ in oocytes is consistent with the physiological observation that the ammonium uptake in maize roots was independent on the extracellular Ca^2+^ (Figure 7D; Supplementary Figure 2). This indicates that the ammonium absorption of maize is resistant to short-term variations in extracellular Ca^2+^. Under our experimental conditions, the ammonium uptake currents of ZmAMT1.1b and ZmAMT1.3 were not affected by the presence of either low (0.05 mM) or high (10 mM) concentrations of nitrate in the ammonium bathing solutions (Figures 5A,C,D). Although the ammonium uptake currents of ZmAMT1.1a remained almost unchanged with the inclusion of lower concentrations (5 mM and below) of nitrate, the current was significantly increased when a high concentration (10 mM) of nitrate was introduced to the testing solution (Figures 5A,B). This resulted in a current increase of 13 to 18 nA (depending on the voltage imposed) compared to the currents measured in the absence of nitrate (Figure 5B). The recording protocol used here allowed highly sensitive and stable capture of current changes as small as 10 nA (Figure 1A; Hao et al., 2020), thus, the nitrate-facilitated effects on ZmAMT1.1a ammonium currents by 10 mM nitrate, in the range of 13 to 18 nA, were statistically significant (Figure 5B).

On the other hand, the observation in oocytes that the presence of nitrate at lower concentrations (below 10 mM) did not have a significant influence on ammonium uptake currents with any of the three ZmAMTs (Figures 5A–D) was in accordance with the physiological measurements in maize roots that the uptake of ammonium was not affected by external nitrate (Figure 7E). Considering that the nitrate concentration in soils is normally within a few millimolars (Bouguyon et al., 2012), the enhanced transport of ammonium by the presence of a high millimolar concentration of nitrate observed with ZmAMT1.1a does not affect the direct uptake of ammonium by roots. Rather, the influence of these high levels of nitrate could instead have a physiological relevance for vascular loading and translocation of ammonium, since in the vascular bundles the presence of nitrate can be as high as 10−30 mM (Andrews, 1986; Wilkinson et al., 2007).

### Mutation of the Highly Conserved “T” to “D” at the Carboxyl-Terminus Exhibits Differential Impacts on the Transport Activity of ZmAMT1s in Oocytes

Evidenced by disappearance of the ammonium-activated currents in the “T” to “D” mutants, the functions of ZmAMT1.1b and ZmAMT1.3 were proposed to be controlled by the potentially conserved phosphorylation site “T460” (Figure 6), which is consistent with the role of corresponding sites in previously reported plant AMTs (Loqué et al., 2007; Neuhäuser et al., 2007; Yuan et al., 2013; Guo et al., 2018; Wu et al., 2019). However, in addition to the loss-of-function after mutation, the absence of ammonium-induced currents in both the T453D-ZmAMT1.1b and T452D-ZmAMT1.3 mutants might also be the result of the inability of mutant transporters to traffic to the plasma membrane. Three observations suggest that this is not the case. First, the equivalent T472D mutation in AtAMT1;2 results in a loss of ability to absorb ammonium, but does not change its localization on the plasma membrane (Neuhäuser et al., 2007). Second, mutations of this “T460” equivalent site to another amino acid, A, lead to the retention of ammonium transport ability in several plant AMTs (Neuhäuser et al., 2007; Guo et al., 2018; Wu et al., 2019), suggesting that mutations at this position do not cause failure to traffic to the plasma membrane. Third, to date, the inability to traffic to the plasma membrane caused by a single point mutation in plant AMTs has not been reported (Ludewig et al., 2003; Neuhäuser et al., 2007). Taken together, the possibility that they fail to traffic to the plasma membrane, thus resulting in the absence of ammonium-induced currents in both T453D-ZmAMT1.1b and T452D-ZmAMT1.3, is very small. These two mutations thus most likely convert ZmAMT1.1b and ZmAMT1.3 into inactive states, in a similar manner as their equivalent mutations from previously reported plant AMTs.

Strangely, the corresponding point mutation of T452D at ZmAMT1.1a did not show a substantial effect on its function in oocytes (Figures 6A,B). A different effect on the transport activity of AMTs resulting from an equivalent point mutation was reported for “H168E”. Most AMTs retain their ammonium transport activity upon the corresponding H168E mutation (Boeckstaens et al., 2008; Ortiz-Ramirez et al., 2011), whereas the equivalent H to E mutation in OsAMT1;3 leads to a loss-of-function (Hao et al., 2016). This reflects the diverse role of this site among AMTs. Here, the substitution of the conserved “T460” with a “D” is proposed to mimic the phosphorylated status of this amino acid site (Loqué et al., 2007; Neuhäuser et al., 2007). Mutation of the equivalent T452D did not affect the transport activity of ZmAMT1.1a (Figure 6), suggesting that the phosphorylated status of this residue does not affect the ammonium uptake functionality of this protein. Similarly, mutation of T452A to mimic its de-phosphorylated status did not regulate the transport activity (Figure 6). These data together suggest that the functionality of ZmAMT1.1a is independent of the phosphorylation status of the T452 residue, at least in oocytes. However, equivalent residues of T452 from other plant AMTs have been proposed to control either the inactivation or activation of the ammonium transporters by their corresponding phosphorylation or de-phosphorylation status (Loqué et al., 2007; Neuhäuser et al., 2007; Yuan et al., 2013; Guo et al., 2018; Wu et al., 2019). The independence of ZmAMT1.1a from the phosphorylation regulation of T452, which is different to equivalent residues from other AMTs, may be the result of the following reasons:

(i) While it is predicted that T452 in ZmAMT1.1a is homologous to the regulatory T460 in AtAMT1;1 (Supplementary Figure 3), it is possible that the regulation of ZmAMT1.1a is achieved through the phosphorylation of a different residue. For example, ammonium ion transport by yeast Mep2, a homolog of plant AMTs, is controlled by phosphorylation of a different site at its carboxyl-terminus, rather than on an equivalent residue to the T460 (Boeckstaens et al., 2014; Van Den Berg et al., 2016).

(ii) In addition to the T460, at least seven phosphorylation sites (S475, S488, S490, S492, T496, T497, and T499) of AtAMT1;1 are identified by phosphoproteomics or phospho-specific antibody analyses, all of which are located downstream of T460 at the carboxyl-terminus (Supplementary Figure 3; Wu et al., 2019). The Y469 stop mutation, which deletes the region containing all these seven phosphorylation sites, does not substantially affect the functionality of AMTs (Loqué et al., 2007; Wu et al., 2019). This suggests that the phosphorylation sites downstream of T460 do not play a significant regulatory role in the transport properties of AMTs. Merely one different residue, which is a non-phosphorylation site, exists in the region between T460 and Y469 among AMTs. Thus, sequences downstream of T460 in the carboxyl-terminus of AMTs do not seem to be important in phosphorylation regulation.

(iii) Concentrating on the T460 upstream region in the carboxyl-terminus, we found a potential phosphorylation site, S447, unique to ZmAMT1.1a (Supplementary Figure 3). It is speculated that the phosphorylation of this site, or the coordination of S447 with the T452 phosphorylation site, could potentially play a role in the functional regulation of ZmAMT1.1a. It has been reported that the function of yeast Mep2, a plant AMT-homolog, is regulated by the phosphorylation status of such an “S”-type residue (S457) at its carboxyl-terminus (Boeckstaens et al., 2014; Van Den Berg et al., 2016). Further research needs to conduct on the phosphorylation effect of the S447 or S447/T452 double sites in ZmAMT1.1a.

(iv) Finally, the identification of the “T460” corresponding site in ZmAMT1.1a was achieved by simple sequence alignment. Given that ZmAMT1.1a has a longer cytosolic carboxyl-terminus than AtAMT1;1 (Supplementary Figure 3), the possibility that T452 might not exactly match “T460” in three-dimensional space could not be ruled out.

The functionalities of the three ZmAMT1s were all suppressed by Gln (Figures 5E−H). Previous studies suggest that Gln feedback inhibits high-affinity ammonium uptake by regulating AMT expression at the transcriptional level (Rawat et al., 1999; Sonoda et al., 2003). The seemingly direct modulation of the function of AMTs by Gln at the protein level presented here might be synergistic with their regulation at the transcriptional level.

## Data Availability Statement

The datasets generated for this study are available on request to the corresponding author.

## Ethics Statement

The animal study was reviewed and approved by the Laboratory Animal Resources, Chinese Academy of Sciences.

## Author Contributions

Y-HS conceived and designed the project and revised the manuscript. D-LH performed the electrophysiology and most of the physiological experiments and wrote the manuscript. J-YZ performed parts of the hydroponic test. D-LH, J-YZ, S-YY, and Y-NH performed the data analyses. All authors contributed to the article and approved the submitted version.

## Conflict of Interest

The authors declare that the research was conducted in the absence of any commercial or financial relationships that could be construed as a potential conflict of interest.

## Footnotes

1 Ammonium is used to refer to the total NH_4_^+^ and NH_3_. The chemical formula NH_4_^+^ represents ionic ammonium, while NH_3_ represents molecular ammonium. This notation is the same as previously that used in a previous study (Ludewig et al., 2002).
