# Supplementary material for: Functional and Regulatory Characterization of Three AMTs in Maize Roots
Source: Front Plant Sci. 2020 Jun 26;11:884. doi: 10.3389/fpls.2020.00884 (PMC7333355; doi:10.3389/fpls.2020.00884)
Supplement: Supplementary file 1 [file Data_Sheet_1.pdf]

# Functional and Regulatory Characterization of three AMTs in Maize Roots

Dong-Li Hao[1], Jin-Yan Zhou[1], Shun-Ying Yang[1], Ya-Nan Huang[1], Yan-Hua Su[1,*]

1 State Key Laboratory of Soil and Sustainable Agriculture, Institute of Soil Science, Chinese Academy of Sciences, Nanjing 210008, China.

* Correspondence:
Yan-Hua Su
yhsu@issas.ac.cn

Word count: 6551 words.

Figures： 8.

**SUPPLEMENTARY MATERIAL**

## Supplementary Figure 1

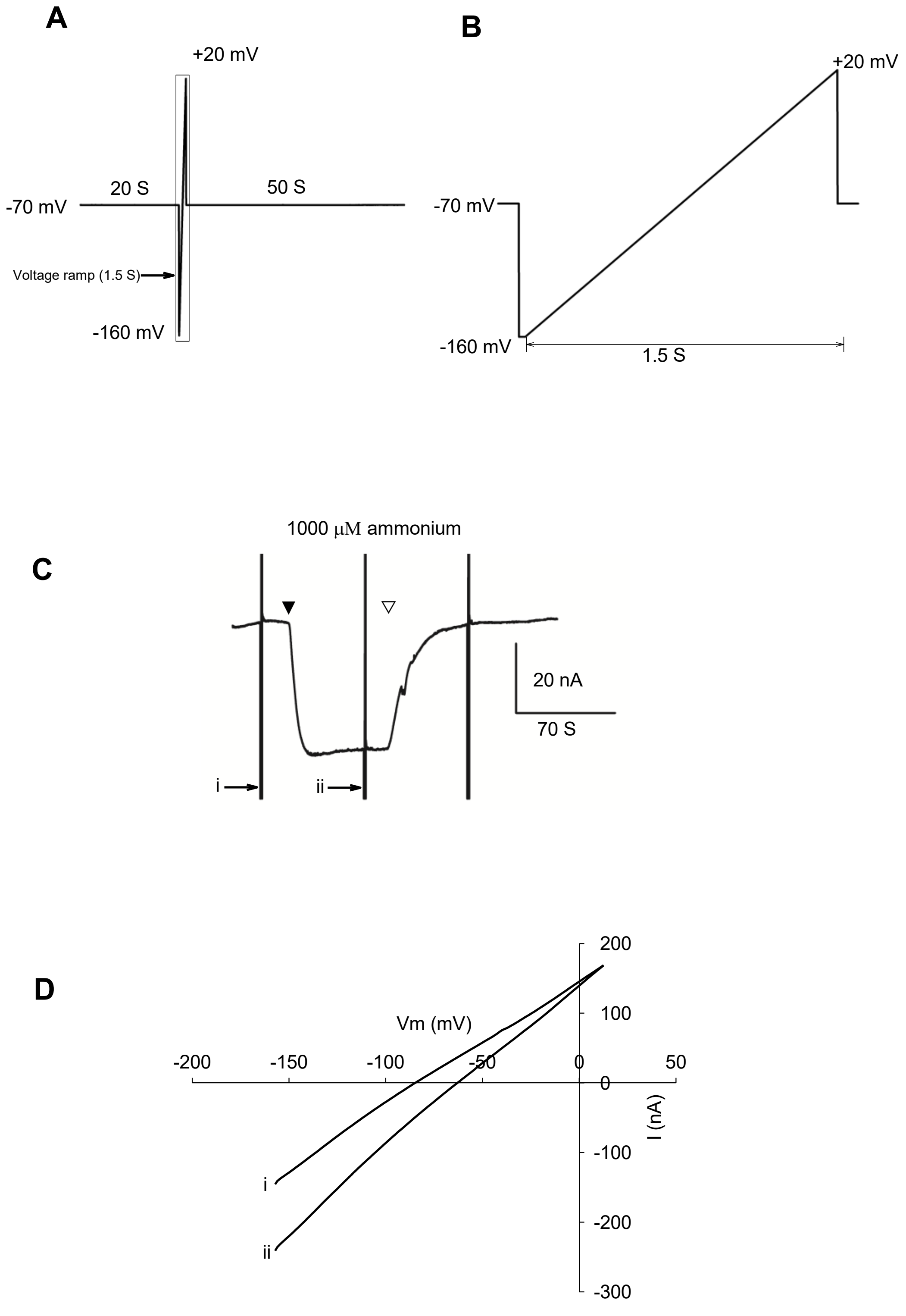

**Supplementary Figure 1. Electrophysiological recording strategy in *Xenopus* oocytes.** **(A)** Recording protocol. The holding potential was fixed to −70 mV, with the exception of a 1.5 s voltage ramp (from −160 mV to +20 mV) applied every 70 s. The box indicated the 1.5 s-long voltage ramp. **(B)** Amplification of voltage ramp in box of A. **(C)** Representative recordings showing the current responses of ZmAMT1.1b-expressing oocytes to 1 mM ammonium. Oocytes were subjected to the ammonium-free solution, followed by 1000 μM ammonium containing solution and a return to the ammonium-free solution. Ammonium was added as a chloride salt. The introduction (▼) or withdrawal (▽) of the ammonium is indicated. The vertical lines were current responses to imposed voltage ramps ranged from −160 mV to +20 mV. **(D)** Current (I)-voltage (Vm) relationship obtained from C. The line i indicated the I-V curve before ammonium addition (in ammonium-free solution), the line ii indicated the I-V curve under the ammonium containing condition.

## Supplementary Figure 2

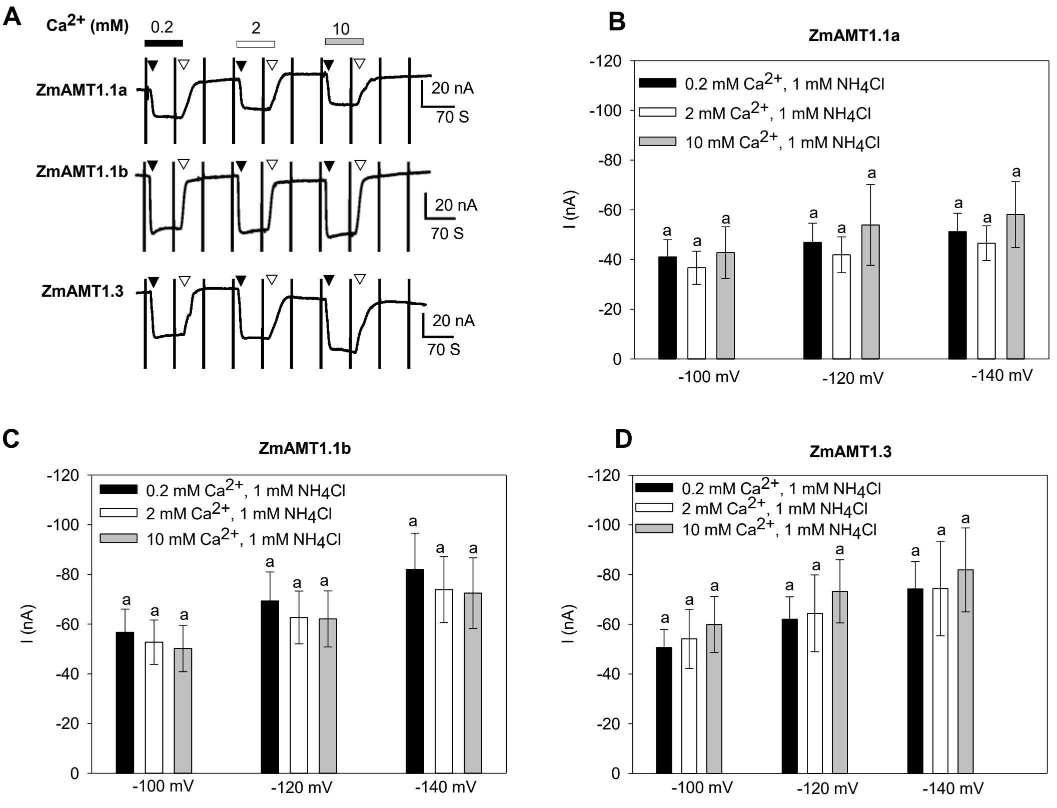


**Supplementary Figure 2. Effect of extracellular $Ca^{2+}$ on the transport activity of ZmAMT1s. (A)** Representative recordings showing the current responses of ZmAMT1s-expressing oocytes to 1 mM ammonium in the co-existence of different concentrations of $Ca^{2+}$. Both the ammonium and $Ca^{2+}$ were added as chloride salts. The protocol used was described in Figure 1. The introduction (▼) or withdrawal (▽) of 1 mM ammonium bath solution differing in concentration of $Ca^{2+}$ is indicated. **(B-D)** Currents induced by 1 mM ammonium in the presence or absence of different concentrations of $Ca^{2+}$ for ZmAMT1.1a **(B)**, ZmAMT1.1b **(C)** or ZmAMT1.3 **(D)** at −100 mV, −120 mV or −140 mV, respectively. No significant differences were detected among $Ca^{2+}$ treatments (LSD, $P < 0.05$). Data shown were mean ± SE (n=3).

## Supplementary Figure S3

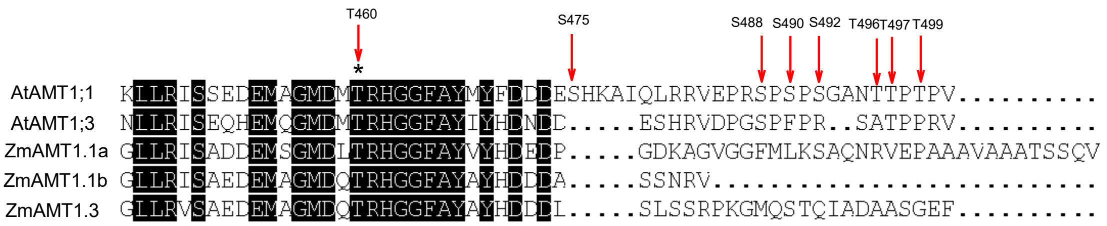


**Supplementary Figure 3. Sequence alignment of ZmAMT1s with AMT1s from Arabidopsis focused on their cytosolic carboxyl-terminus region.** Eight phosphorylation sites in AtAMT1;1 were indicated by red arrow. * indicated the conserved "T460". The "T464" site in AtAMT1;3, the "T452" site in ZmAMT1.1a, the "T453" site in ZmAMT1.1b, and the "T452" site in ZmAMT1.3 correspond to the T460 site in AtAMT1;1. The protein accession numbers are listed below: AtAMT1;1 (1703292), AtAMT1;3 (5880355), ZmAMT1.1a (GRMZM2G175140_P01), ZmAMT1.1b (GRMZM2G118950_P01), and ZmAMT1.3 (GRMZM2G028736_P01).

**Supplementary Table 1. Primers used for real-time quantitative PCR.**

| Gene | | Primers (5'-3') |
|---|---|---|
| *ZmAMT1.1a* | qF | AAGCTCCTCGCCGCGCAGA |
| | qR | GGCCTTGTCGCCAGGGTCCTC |
| *ZmAMT1.1b* | qF | AAGCTCCTGGCCGCGCAGATCG |
| | qR | TGCATTGTCGCCAGGGTCCTC |
| *ZmAMT1.3* | qF | AGGCTGCTGGCCGCGAACGTGG |
| | qR | AAGTCGTCGTCGTGGTACGCG |
| *ZmACT1* | qF | GGAGATCACGTCCCTGGCTCC |
| | qR | CTCTCCCTTGGAGATCCACATC |

**Supplementary Table 2. Primers used for vector construction.**

| Gene | | Primers (5'-3') |
|---|---|---|
| *ZmAMT1.1a* | F | cgagaattcacgcgtggtaccATGTCGACGTGCGCGGCG |
| | R | tcatgtctgctcgaagcggccgcTTACACCTGGCTGCTGGTCG |
| *ZmAMT1.1b* | F | cgagaattcacgcgtggtaccATGGCGACGTGCGCGACG |
| | R | tcatgtctgctcgaagcggccgcTTACACCCGGTTGCTGCTCG |
| *ZmAMT1.3* | F | cgagaattcacgcgtggtaccATGGCGACGTGCGCTACG |
| | R | tcatgtctgctcgaagcggccgcCTAGAACTCGCCGCTGGCC |
| *T452D-ZmAMT1.1a* | P2 | GAAGCCGCCGTGCCGGTCCAGGTCCATGCCG |
| | P3 | CGGCATGGACCTGGACCGGCACGGCGGCTTC |
| *T452A-ZmAMT1.1a* | P2 | GAAGCCGCCGTGCCGGGCCAGGTCCATGCCG |
| | P3 | CGGCATGGACCTGGCCCGGCACGGCGGCTTC |
| *T453D-ZmAMT1.1b* | P2 | GAACCCGCCGTGGCGGTCCTGGTCCATGCCG |
| | P3 | CGGCATGGACCAGGACCGCCACGGCGGGTTC |
| *T452D-ZmAMT1.3* | P2 | GAACCCGCCGTGCCGGTCCTGGTCCATGCCG |
| | P3 | CGGCATGGACCAGGACCGGCACGGCGGGTTC |
| *AtAMT1;3* | F | cgagaattcacgcgtggtaccATGTCAGGAGCAATAACATGCTCT |
| | R | tcatgtctgctcgaagcggccgcTTAAACGCGAGGAGGAGTAGCT |
| *T464D-AtAMT1;3* | P2 | GCCACCGTGACGGTCCATATCCATCC |
| | P3 | GGATGGATATGGACCGTCACGGTGGC |

**Note:** phosphorylation site mutants were constructed using overlapping PCR. For example, as to T452D-ZmAMT1.1a mutant, first round of PCR: using *ZmAMT1.1a*-pCI as the template, ZmAMT1.1a-F and T452D-ZmAMT1.1a-P2 pair, T452D-ZmAMT1.1a-P3 and ZmAMT1.1a-R pair, respectively as primers, to obtain two PCR products. The T452D-ZmAMT1.1a mutant was then obtained by a second round of PCR using ZmAMT1.1a-F and ZmAMT1.1a-R as primers, mixed PCR products from first round as the template.
